# Evaluation of novel formulations for transarterial chemoembolization: combining elements of Lipiodol emulsions with Drug-eluting Beads

**DOI:** 10.7150/thno.34778

**Published:** 2019-07-28

**Authors:** Marcus Caine, Ting Chung, Hugh Kilpatrick, Zainab Bascal, Sean Willis, Yiqing Tang, Thierry de Baere, Matthew Dreher, Andrew Lewis

**Affiliations:** 1 Biocompatibles UK Ltd (a BTG International group company), Lakeview, Riverside Way, Watchmoor Park, Camberley, Surrey, GU15 3YL, UK.; 2 Gustave Roussy 114, rue Edouard Vaillant, 94805, Villejuif, France

**Keywords:** Transarterial chemoembolization, Drug-loaded emulsions, *in vitro-in vivo* correlation.

## Abstract

There are currently two methods widely used in clinical practice to perform transarterial chemoembolization (TACE). One is based on mixing an aqueous drug with an iodized oil (Lipiodol) and creating an emulsion that is delivered intraarterially, followed by embolization with a particulate agent. The other is based on a one-step TACE using Drug-eluting Beads (DEBs) loaded with drug. It is not recommended to mix Lipiodol with DEBs due to incompatibility. For the first time, novel DEB: Lipiodol: doxorubicin (Dox) emulsions are identified using lyophilized polyvinyl alcohol (PVA) hydrogels (non-iodinated or iodinated) DEBs.

Methods: 15 DEB emulsions (50mg Dox) were assessed for stability and deliverability *in vitro* and *in vivo* in a swine model. Dox release from selected formulations was measured *in vitro* using a vascular flow model and *in vivo* in a VX2 rabbit tumor model.

Results: Both DEB formats were shown to be able to form emulsions, however only Iodinated DEBs consistently met defined handling criteria. Those based on the non-iodinated DEB achieved >99%+ Dox loading in <5 minutes but were generally less stable. Those prepared using iodinated DEBs, which are more hydrophobic, were able to form stable Pickering-like emulsions (separation time ≥ 20 minutes) and demonstrated handling, administration and imaging observations more akin to Lipiodol™ TACE emulsions in both embolization models. Controlled Dox release and hence beneficial *in vivo* pharmacokinetics associated with DEB-TACE were maintained.

Conclusions: This study demonstrates that it is possible to formulate novel DEB emulsions suitable for TACE that combine positive elements of both Lipiodol™ based and DEB-TACE procedures.

## Introduction

The theory behind conventional Lipiodol Ultra Fluid™ (Guerbet, France) based transarterial chemoembolization (TACE, or cTACE) is founded on the premise that a water-in-oil (w/o) emulsion can be formed when Lipiodol™ is mixed with an aqueous drug-containing solution at an appropriate oil:water ratio 1. When the emulsion is delivered through a microcatheter into the flowing blood stream, viscous oil droplets form that follow the flow into the tumor microvasculature where the viscosity causes cessation or slowing of flow into the small vessels. The chemotherapeutic is trapped in the aqueous compartment of the emulsion and is released as the oil becomes entrapped in the capillary bed. An additional embolic agent is desirable to induce stasis and prevent the oil being washed through prematurely and increase its dwell time. A proportion of the droplets are of a size that can pass through the sinusoids into the portal venous system 2, 3 and greater volumes of the liver can therefore be exposed to the chemotherapeutic than intended. The Lipiodol™ is eliminated from the healthy part of the liver by the action of Kupffer cells 4 but as liver tumors do not possess these, the area within the tumor remains stained by the Lipiodol™ appearing as a dense radiopaque region on follow-up X-ray based imaging 3.

Drug-eluting Beads (DEBs) were introduced over a decade ago with the intention of standardizing TACE by providing calibrated microspheres that contain the chemotherapeutic agent that may be used to embolize the vessels of the tumor where they then elute the drug in a locoregional manner, over a number of days to weeks 5. It has been demonstrated in clinical studies that plasma levels of drug are significantly reduced when comparing DEB with Lipiodol™ TACE, resulting in fewer side effects, less liver toxicity and shorter hospital stays 6-8. Whilst quality of life may therefore be improved, there is still considerable debate as to whether there is any benefit in overall survival for DEB *versus* cTACE. Indeed, over the years one practice has not superseded the other, physicians choosing one or the other based upon the patients overall health, size and number of tumors, whether discrete or diffuse and their location in the liver 9-12.

Whilst there are anecdotal reports that some physicians use mixtures of DEBs and Lipiodol™, this is not currently recommended 10 and there are no accounts in the literature to verify this practice. DEBs are suspended in aqueous iodinated contrast agents in order that the suspension can be visualized under fluoroscopic administration. DEBs are highly hydrophilic and if mixed with oil, remain dispersed in the aqueous phase, the admixture disrupting the emulsion stability creating a concoction unsuitable for delivery. A recent study reported on the use of oxaliplatin-loaded polylactide-glycolide copolymer nanoparticles to form stable emulsions with Lipiodol™ suitable for TACE 13. These relatively hydrophobic nanoparticles adsorb at the water-oil interface and sterically stabilize the droplets preventing coalescence, forming a system known as a Pickering emulsion 14. Whilst the nanoparticles also acted as a controlled release system for the oxaliplatin, their size was too small to provide for concomitant embolization of the vessels. DEB:Lipiodol™ emulsions therefore present a new paradigm for TACE that currently remains un-investigated. In order to overcome the incompatibility issues experienced with current DEB: Lipiodol™ emulsions and achieve Pickering-like stability, the particles need to have the appropriate balance of hydrophilic and hydrophobic character. This study presents two strategies to enhance the hydrophobic nature of the DEBs and evaluates the stability, deliverability, coherence under flow and drug delivery properties both *in vitro* and *in vivo* of the subsequent novel DEB:Lipiodol™ stabilized TACE emulsion formulations.

## Materials and Methods

### Preparation of non-iodinated and iodinated PVA hydrogel DEBs

The two DEB types prepared for this study were a non-iodinated DEB based on the PVA-hydrogel used in the fabrication of DC Bead™ 15 (Figure 2A) and an iodinated DEB (containing 155 mg iodine/mL) based on the PVA-hydrogel used in the fabrication of DC Bead LUMI™ 16 (Biocompatibles UK Ltd, Farnham, UK (Figure 2B). A second iodinated DEB (containing 33 mg iodine/mL) was also prepared for a Contact Atomic Force Spectroscopy (CAFS) study only. Non-iodinated PVA beads were immersed in 10% mannitol solution for 30 minutes and excess solution removed prior to lyophilization in order to prevent collapse of the internal structure (96% water content). The iodinated PVA beads did not require this step as they possess higher solid content (~65% water content). Comparable size ranges of each bead type (70-150 µm) were frozen (-20°C) for 30 minutes and then lyophilized (Wizard 2.0, VirTis, SP Scientific, USA) for 18 hours until visibly dry and free flowing particles were observed (as described previously for the non-iodinated PVA DEBs 17). This lyophilized format allows for initial mixing with Lipiodol™ oil which can penetrate the DEBs structure and aid in the emulsion compatibility (Figure 2A step 3).

### DEB surface property evaluation

Initial qualitative assessments were performed on a Dimension 3000 atomic force microscope (AFM) (Digital Instruments) and data collected using the NanoScope IIIA software (Digital Instruments) 18. The force distance curve was obtained by measuring the deflection signal (voltage) from the cantilever as the probe approached the surface from approximately 750-1800nm above at a constant scan rate of 1Hz. The cantilever deflection was observed as the difference in signal voltage at the points when the tip is retracting from the particle surface and when the tip is free from the surface. Using a silicon AFM probe, it was expected that the adhesion and cantilever deflection would be highest in particles having a more hydrophilic surface property. Testing was performed in n= 3 replicate samples. The cantilever deflection was measured as a signal voltage and was calculated to give an adhesion force using the spring constant of the cantilever.

### DEB:Lipiodol™ emulsion formulation

Emulsions (containing DEBs) with different oil and water ratios were formulated by the mixing of proportionate volumes of Lipiodol Ultra Fluid™ (Guerbet, France) and aqueous solution, in the presence of the DEBs. The aqueous solution was comprised of doxorubicin hydrochloride (Dox, Hisun, China)) solution and aqueous Omnipaque 350™ contrast agent (GE Healthcare, UK). The overall ratios of oil to aqueous investigated ranged from 53:47%v/v to 83:17%v/v, the volume of the DEBs were not expressed in the Oil:Water ratio as this was fixed at 2mL. Both iodinated and non-iodinated DEBs were analysed over this range.

Depending on the desired oil:water ratio, an amount of Lipiodol™ (Guerbet, France) was added directly to vials of each bead type and the sample allowed to stand for 5 minutes before aspirating into a 20 mL syringe. The desired amount of an aqueous Dox solution (50 mg total Dox) was added to a 10 mL syringe and if required, a further volume of aqueous contrast agent was added to this to increase the density of the aqueous phase and further improve stability of the emulsion. The emulsion was formed by purging the aqueous contents of the 10mL syringe into a 20 mL syringe *via* a 3 way connector, and then back again into the original 20 mL syringe. This was repeated 10 times, i.e. a total of 20 passes of the mixture through the 3-way connector. The emulsion was left to stand for up to 60 minutes (with resuspension of the emulsion every 10 minutes) to allow drug load into the DEBs and was subsequently resuspended one final time before being analysed for emulsion stability or flow properties.

**Figure 1:**
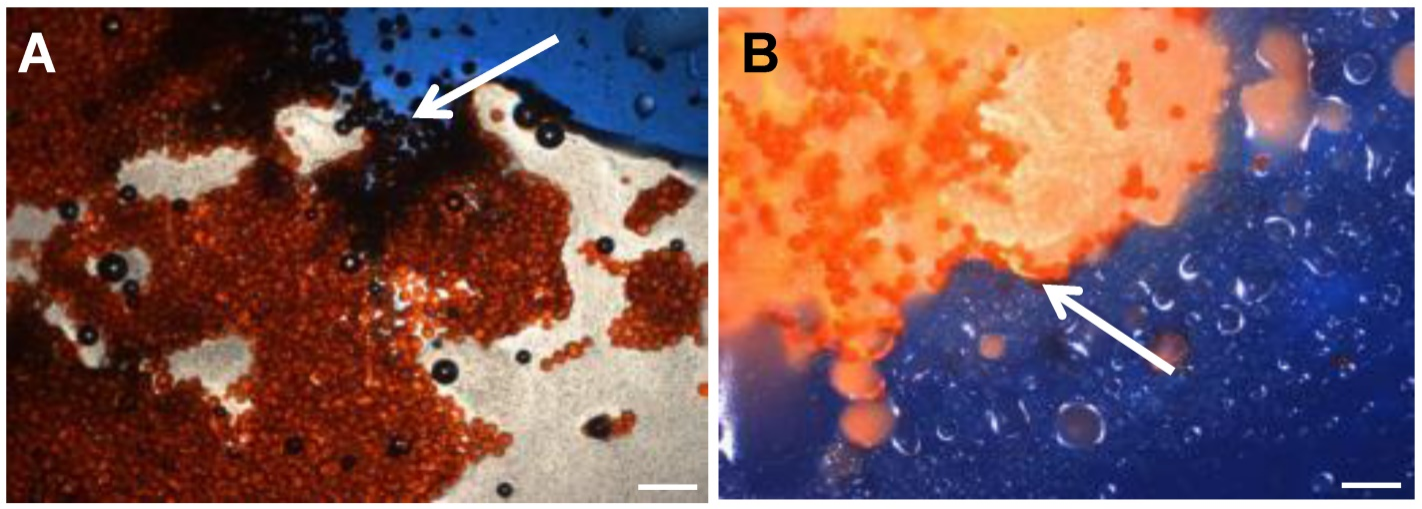
Optical microscopy of DEB Emulsions. Arrow A shows water soluable indicator dye (Reactive Blue 4,. Sigma Aldrich) entering the aqueous phase containing drug loaded beads in Oil:Water emulsion, generally described as less stable under flow. Arrow B indicates no contract with beads suspended in Water:Oil emulsion, generally defined as more stable under flow (4x Mag, BX50 Olympus). Scale bar 200µm.

Visual light microscopy of the emulsions was performed using a microscope at 10x magnification (BX50, Olympus, Japan) and using methods adapted from Pichot et al. 19. Emulsions were prepared and dispensed into a petri dish, a cover slip added (22x22 mm Manzel-Glaser, Avonchem LtD, UK) and then a drop of aqueous blue dye (Reactive Blue 4, Sigma Aldrich) was applied to the edge of the cover slip to identify the aqueous phase and hence the preferred location for the DEBs as shown in the micrographs in Figure 1. In some experiments the emulsion was sampled periodically to determine the extent of Dox loading into the DEB by spectrophotometric determination of the residual drug remaining 15 and visual confirmation of loading by light microscopy.

### cTACE emulsion formation

cTACE emulsions were prepared by direct mixing of proportionate volumes of Lipiodol and aqueous doxorubicin hydrochloride solution. The emulsion was formed by purging the aqueous doxorubicin solution (10mL syringe) into the Lipiodol (20mL syringe) via a 3 way connector, and then back again 10 times. Although in clinical practice this would be followed up with an embolic particle to finalise the embolization, we attempt to show the burst effect of drug associated with purely the emulsions, prior to any additional embolisation.

### DEB Emulsion stability evaluation under static and flow conditions

#### DEB Emulsion separation time

Emulsion stability was first assessed by recording the time it took for phase separation to become evident in the syringe immediately after it had been mixed through the 3-way connector. Separation time was taken as the onset of inhomogeneity in emulsion appearance as distinct layers began to form as the phases separated (see Figure 3D).

#### DEB Emulsion drop test

DEB emulsions were injected in a steady stream *via* an 18G blunt fill needle (BD, US) into saline solution in an adaptation of the pendant method presented by Pichot *et al.* 19. The droplet integrity and formation of strings were evaluated as a measure of emulsion stability and cohesion in response to limited shear and aqueous contact. Good emulsions were classified as having discreet droplets that remained intact following delivery from the needle as visually observed (Figure 5B& E). Poor indicators of stability were defined as streams of loose DEBs and separated oil droplets containing few or no DEBs (Figure 5A&D). Assessments were repeated a minimum of 3 times for each formulation.

#### DEB Emulsion performance assessment under dynamic flow in a Vascular Flow Model

In order to evaluate DEB emulsions under flow, a modified set-up using a previously described Vascular Flow Model (VFM) was employed 20. Emulsions were injected into flowing saline solution under various clinically representative flow rates, with the catheter (2.4Fr Progreat, Terumo Tokyo) positioned in a location that was agreed to be an approximation of a typical injection site arterial lumen diameter (channel diameter between 2 mm-4 mm). This was confirmed visually with the assistance of an interventional oncologist with >20 years' experience in TACE procedures and who had also previously used similar versions of the flow model to evaluate TACE emulsion behaviour 21. Each emulsion was repeated a minimum of 3 times. The rate was selected as visually representative of blood flow observed under fluoroscopy ranging from 120 mL/min, periodically reduced to 60-30 mL/min to recreate the effect of embolization and flow restriction. The site of the injection was varied between a distal and proximal location (Distal= 2 mm channel diameter, proximal= 4 mm channel diameter) to visually assess the influence of channel aspect ratio, shear rate and flow rate of the fluid surrounding the catheter tip. Optical video microscopy (Dinolite digital microscope (AnMo Electronics Cop. Taiwan) was also used to record DEB emulsion behaviour under flow conditions in the VFM (Videos in supplemental S1). An in-line pressure transducer (Research grade BP, Harvard instruments, UK) was linked to real-time flow measurements during delivery as a measure of the embolic effect of the emulsions on flow (Figure 5C).

#### Grading of DEB emulsions

DEB emulsion performance was graded (1* (poorest) - 5* (best)) based upon the results from the separation time, drop test and flow properties (Table 2). Together these provide a measure of the ability of the microspheres to stabilize the oil:water interface and influence the oil droplet size (streaming or discreet droplet formation) and integrity (its tendency to breakup under flow). Such factors also affect the resistance experienced during delivery through the microcatheter, which is also a predictor of whether the formulation would be suitable for a TACE procedure.

### Drug elution properties of DEB emulsions

For Dox elution determination, the microcatheter was positioned into a single channel of the VFM, covered with a 25 μm microporous mesh (Sefar AG, Switzerland) at the outlet to prevent DEB passage and allow accumulation of an occlusion (Figure 6A). The flow was set to 120 mL/min as per previous flow studies 22, and injection rate was determined to be 0.5 mL/min from previous emulsion bench testing. The eluent was collected from this outlet, starting from the initiation of the DEB delivery and every minute for the first ten minutes then every five minutes until a 30 minute test period was complete. Dox concentration in the eluent was determined using UV-visible spectrophotometry (Varian CaryBio50, UV/Vis Spectrophotometer) set at an absorbance maximum of 483 nm and an elution curve was constructed from the data. The experiment was repeated 3 times for each emulsion tested.

### *In vivo* evaluation of DEB emulsions

Animal testing was performed in accordance with the French regulation regarding experiments conducted on live animals (Decree No. 2013-118 dated 2013 February 1st from the French Ministry of Food and Forestry, on the protection of animals used for scientific purposes, adapting the Directive 2010/63/EU of the European Parliament and of the Council dated 2010 September 22nd on the protection of animals used for scientific purposes). The protocols were reviewed by the local ethics committee (Comite Regional d'Ethique applique a l'Experimentation Animale du Centre INRA de Jouy-en-Josas et d'AgroParisTech, COMETHEA, registered under ID n°45) and approved by the Ministere de l'Enseignement Superieur et de la Recherche (MESR).

#### Drug elution properties in rabbit VX2 model

New Zealand white rabbits (N=7) aged more than 5 months weighing between 3 and 4 kg were implanted with VX2 donor tissues and grown in tibial cranial muscle. Following maturation, 4-5 mm sized tumor sections were harvested and transplanted directly into test liver parenchyma (7 animals per test arm) and allowed to further implant for 12-14 days.

Vascular access to the right femoral artery was performed surgically after incision of the hindmuscle, exposure of the femoral artery and insertion of a 4 French vascular sheath. Selective catheterization of the hepatic artery and super-selective catheterization of the left branch of the hepatic artery was performed using a 1.7 Fr microcatheter under fluoroscopy guidance (Discovery IGS 730, GE Healthcare). An angiogram and a 3D C-arm CBCT imaging was performed and recorded to visualize the tumor vasculature. The embolic agent was slowly administered under fluoroscopy monitoring with care being taken to avoid reflux in non-catheterized arteries. The endpoint for injection was fixed at 1mg Dox as calculated from the formulation composition in all the groups. If stasis of the tumor-feeding artery is not reached after injection of the formulation, bland particles were injected to complete the devascularisation. Due to anatomical restrictions a smaller size of radiopaque DEBs in the range 40-90 µm (Biocompatibles UK Ltd, Farnham, UK), were employed in VX2 model, these were also utilised in the swine model for handling comparison for both iodinated and non-iodinated. A control angiography and a 3D C-arm CBCT imaging was performed after embolization and after 7 days.

Qualitative comparisons of proportional drug release and bio-distribution were compared to *in vitro* observations and quality indicators for a measure of the predictive capabilities of the profiling experiments. Plasma analysis was performed at T= 0 min, 5 min, 15 min, 30 min, 60 min, 2 h, 6 h, 24h, 72 h and 7 days after the end of injection (10 samples per animal). Tissue analysis was performed at 7 days after injection in the tumor and in the peritumoral healthy parenchyma (2 samples per animal).

#### DEB emulsion handing and *in vivo* flow properties in healthy swine model

A total of 4 swine (FBM minipigs) were used for the study (2 female and 2 castrated males). Animals were aged 7 months with a mean weight of 38.7 ± 4.5 kg (min-max 34 - 44 kg). Each animal was identified by a unique national identification number attached to the ear. The number of the animal was verified before anaesthesia and before transfer to the angiography suite. A right femoral artery puncture was performed percutaneously and a 5 French vascular sheath was placed. Alternatively, a surgical access to the femoral artery was gained after incision of the hind muscle and exposure of the femoral artery for one animal. The angiography suite was equipped with a Discovery IGS 730 Image Guided System (GE Healthcare, Little Chalfont, UK). A 5 French catheter (Glidecath® vertebral, Terumo, Guyancourt France) with a 0.035'' angled hydrophilic wire (Terumo, Japan) was used to reach the celiac artery. A super-selective catheterization of one branch of the hepatic artery was performed using a 2.4 French Progreat® microcatheter (Terumo, Japan) and a 0.016” 45° GT wire (Terumo, Japan). The formulation volume prepared depended upon the ratio of the various components (see Table 4) and was between 7.5 mL and 20 mL. When ready for testing, 0.5 mL of formulation was transferred into a 1 mL syringe for ease of delivery. The formulation was administered as intermittent small “puffs” and the 1mL syringe recharged with the resuspended emulsion once emptied, until complete stasis of the catheterized arterial territory was achieved or injection of the full volume of formulation prepared. The microcatheter was then moved to a second hepatic artery branch and a second formulation was injected with the same endpoint. Selective catheterization of the renal artery or of the upper/lower branch of the renal was performed successively in each kidney and the same formulations as in the liver were injected until complete stasis. All animals were sacrificed at the end of the embolization procedure by intravenous injection of 20 mL pentobarbital (Dolethal® 200 mg/mL, Vetoquinol, Lure France) followed by exsanguination. Liver and kidneys were explanted and fixed in 10% formalin. All sequences of injection were recorded. 3D-CBCT sequences were recorded after every 1.0 mL injection.

Emulsions tested included iodinated and non-iodinated beads of two sizes across a range of oil:water ratios (Table 4), selected to cover the working range identified in Figure 5, Table 3. The following parameters were graded by the interventional radiologist (who had been blinded to the formulation compositions), in order to provide an overall handling rank: Syringe stability, Catheter delivery, Radio-opacity, Stability of emulsions during injection, portal opacification during injection, degree of embolization and distality of embolization.

## Results

### Surface characterization of non-iodinated and iodinated PVA hydrogel DEBs

**Figure 2:**
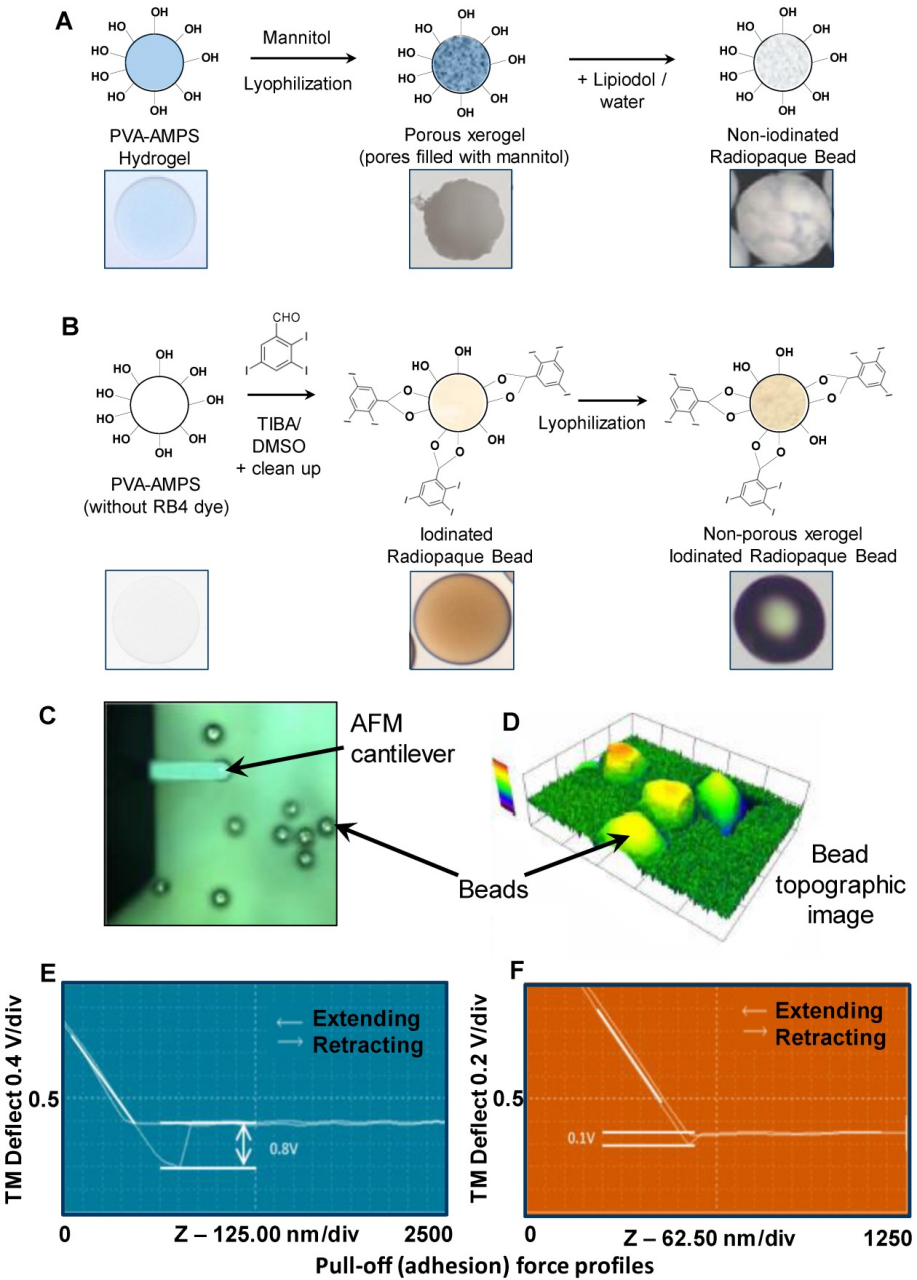
DEB core chemistry for Non-iodinated (A) and Iodinated DEBS (B) resulted in variations in surface hydrophobicity that were characterised using a CAFS system (C) to create topographic (D) and average pull-off force in mV (E/F) across a range of non-iodinated and iodinated DEBs (G).

Measurements were performed on the two DEB types using CAFS and force-distance-amplitude protocols (see Methods, Figure 2C & D) to gain an appreciation of the comparative surface hydrophilicity/hydrophobicity of the DEBs. The force-amplitude-distance measurement for iodinated *versus* non-iodinated DEBs varied (see Figure 2E & F and Table 1), suggesting their surface chemistries were different and that they may behave differently when formulated into emulsions.

### DEB emulsion stability under static conditions

**Figure 3:**
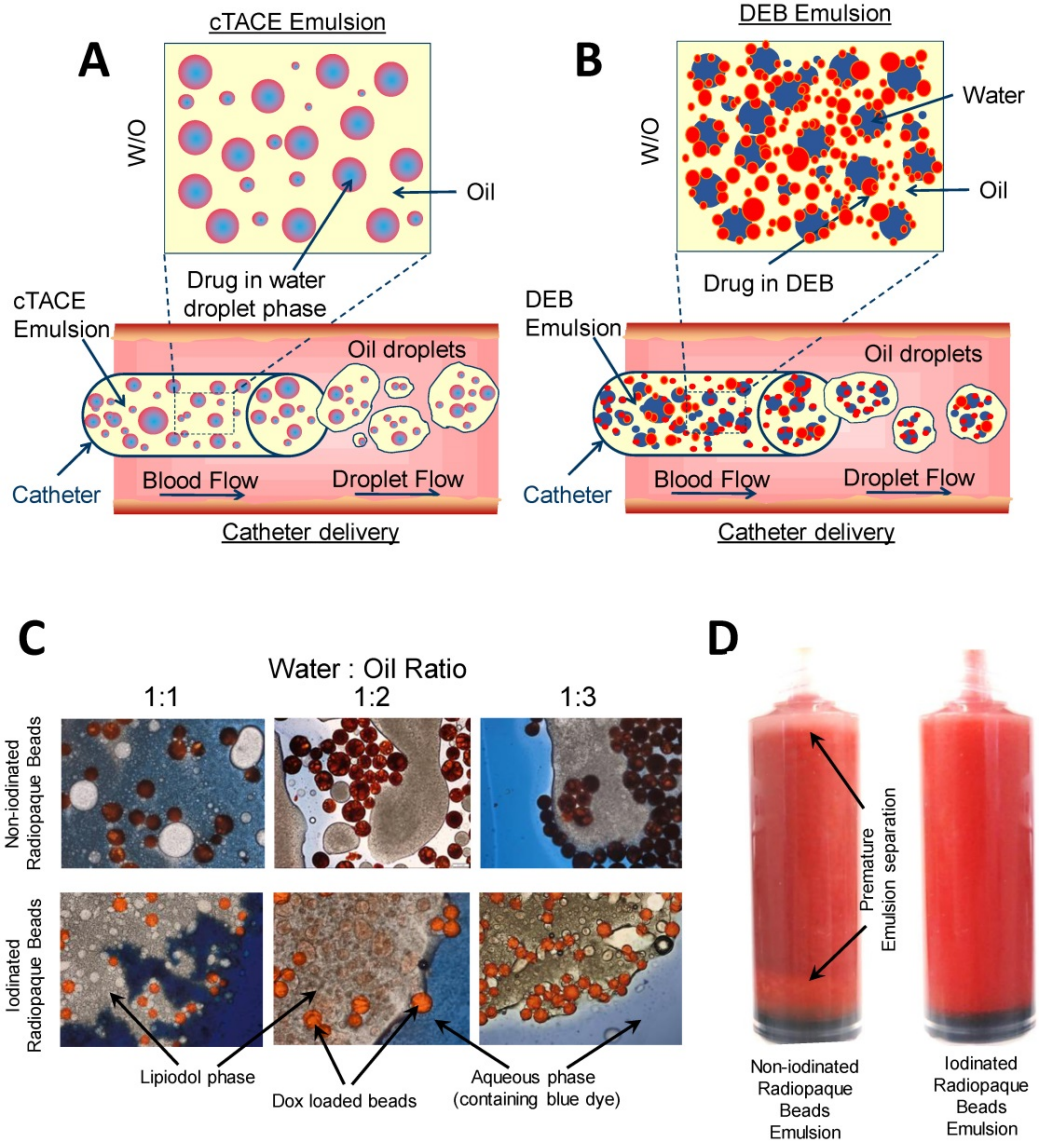
Graphical representation of; cTACE (A) and modified bead comtaining DEB emulsion (B) to show the residence of drug loaded beads for controlled release. Modifications of oil: water ratios and DEB chemistry influenced emulsion stability, DEB oil:water residence (C) and suspension time (D).

Lyophilization of the DEBs allowed for mixing with Lipiodol™ oil without premature separation. In a Lipiodol™ cTACE emulsion, sufficient oil phase is present in the emulsion formulation such that aqueous drug droplets are confined within the oil phase, the latter subsequently forming oil droplets within the continuous aqueous-based blood phase during catheter delivery (hence forming a water-in-oil-in water emulsion (w/o/w) in the body, Figure 3A). The objective of modifying the DEB surface properties was to encourage the DEBs to reside at the oil:water interface and to stabilize the emulsion droplets (Figure 3B). Optical microscopy of the DEB emulsions showed non-iodinated DEBs to have a greater affinity for the aqueous phase of the emulsion, whereas the iodinated DEBs were found to reside more preferentially in the oil phase and at the oil:water interface (Figure 3C). Optical microscopy also confirmed that the Dox was taken up into both DEB types preferentially, rather than residing in the aqueous compartment of the emulsion. Interestingly, Dox uptake from the emulsion containing the non-iodinated DEB was extremely rapid (>98% loaded in <2 mins) compared to the equivalent size of DC Bead prepared by the recommended hospital pharmacy loading methods (>98% in 30 mins). There was no change however, in the rate of Dox uptake from the emulsion into the more hydrophobic iodinated DEB compared to the equivalent size of DC Bead LUMI prepared by standard loading methods (>98% loaded in ~90 mins). Stability of the DEB emulsions in a syringe was defined as an important quality parameter (Figure 3D). The time taken for the emulsions to separate in the syringe was assessed and used to generate an overall ranking combined with static and flow stability observations (Table 2). This ranking was used to decide which formulations to take forward for further evaluation.

**Figure 4:**
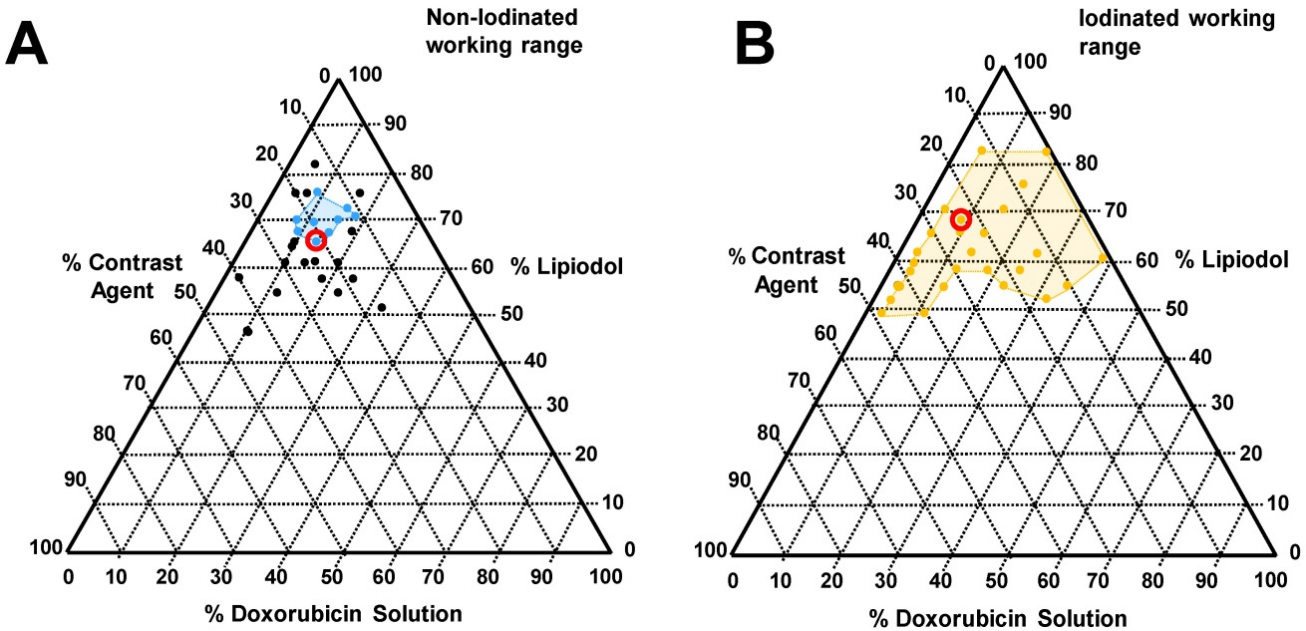
Tested emulsions range with corresponding phase diagram indicating identified favourable working ranges (coloured lines in phase diagram, A, Blue: non-iodinated beads and B, gold: Iodinated beads). Formulations circled within the phase diagram were selected for incorporation into the VX2 drug relase experiments for *in vivo* comparison.

A wide range of emulsion formulations were prepared and evaluated (Table 3). Stable o/w emulsions were observed more prominently in the iodinated DEB samples and with low aqueous content (Table 3 and Figure 4). The iodinated DEB samples indicated partition preference into the oil phase, whereas the non-iodinated samples remained in the aqueous phase, even in low aqueous ratio emulsions. There was increased presence of non-iodinated DEBs at the oil:water interface in low aqueous content, which helped to stabilize these emulsions compared to those with higher aqueous content. This is clearly observed in Figure 3C under the microscope for 1:1 *versus* 1:3 water:oil ratios.

### DEB emulsion stability under flow conditions

**Figure 5:**
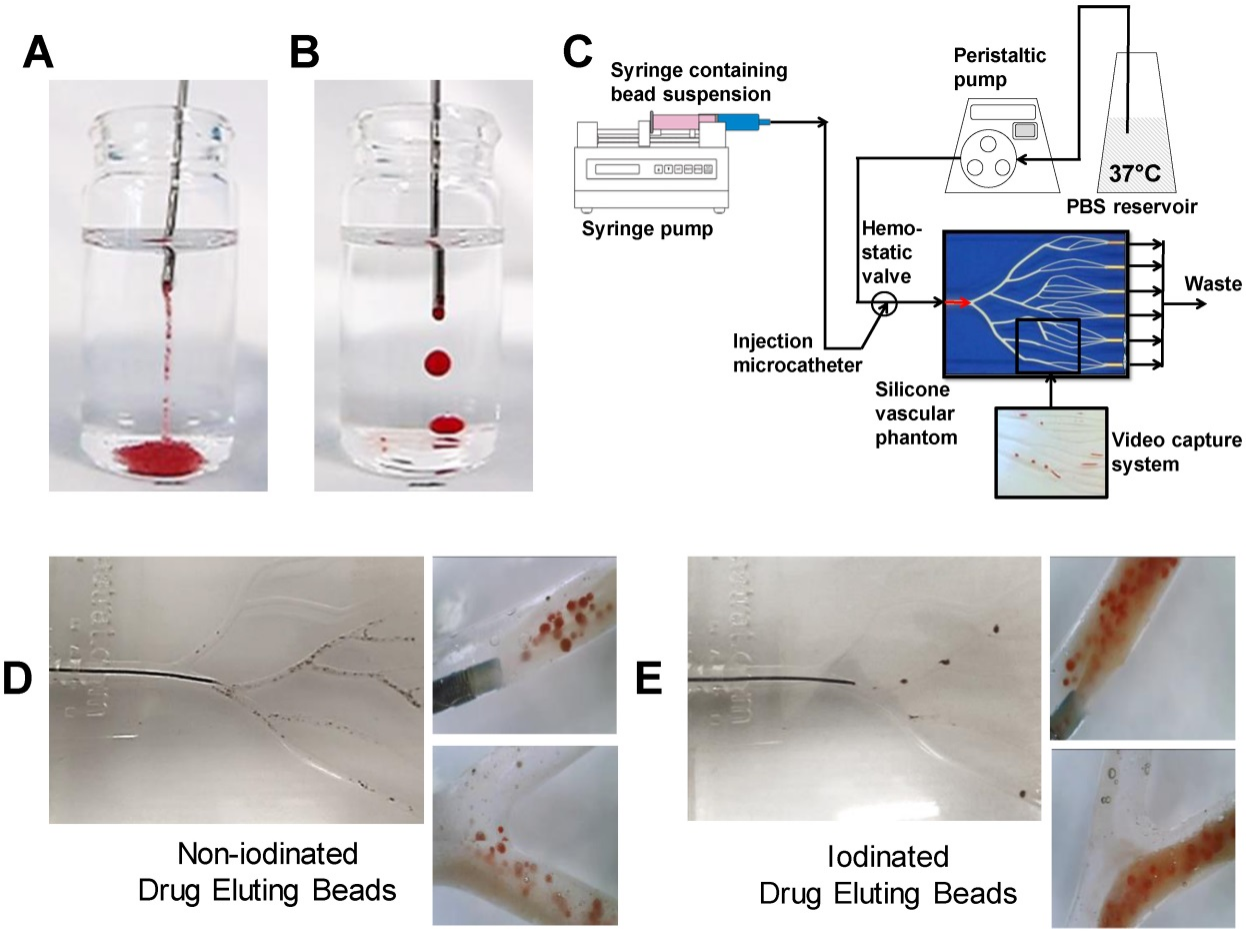
Non-iodinated (A) and Iodinated (B) pendant drop test showing intact droplet formation. Dynamic flow profiling in the VFM (C) utilising video capture to evaluate emulsion flow properties for each bead type (D/E).

**Figure 6:**
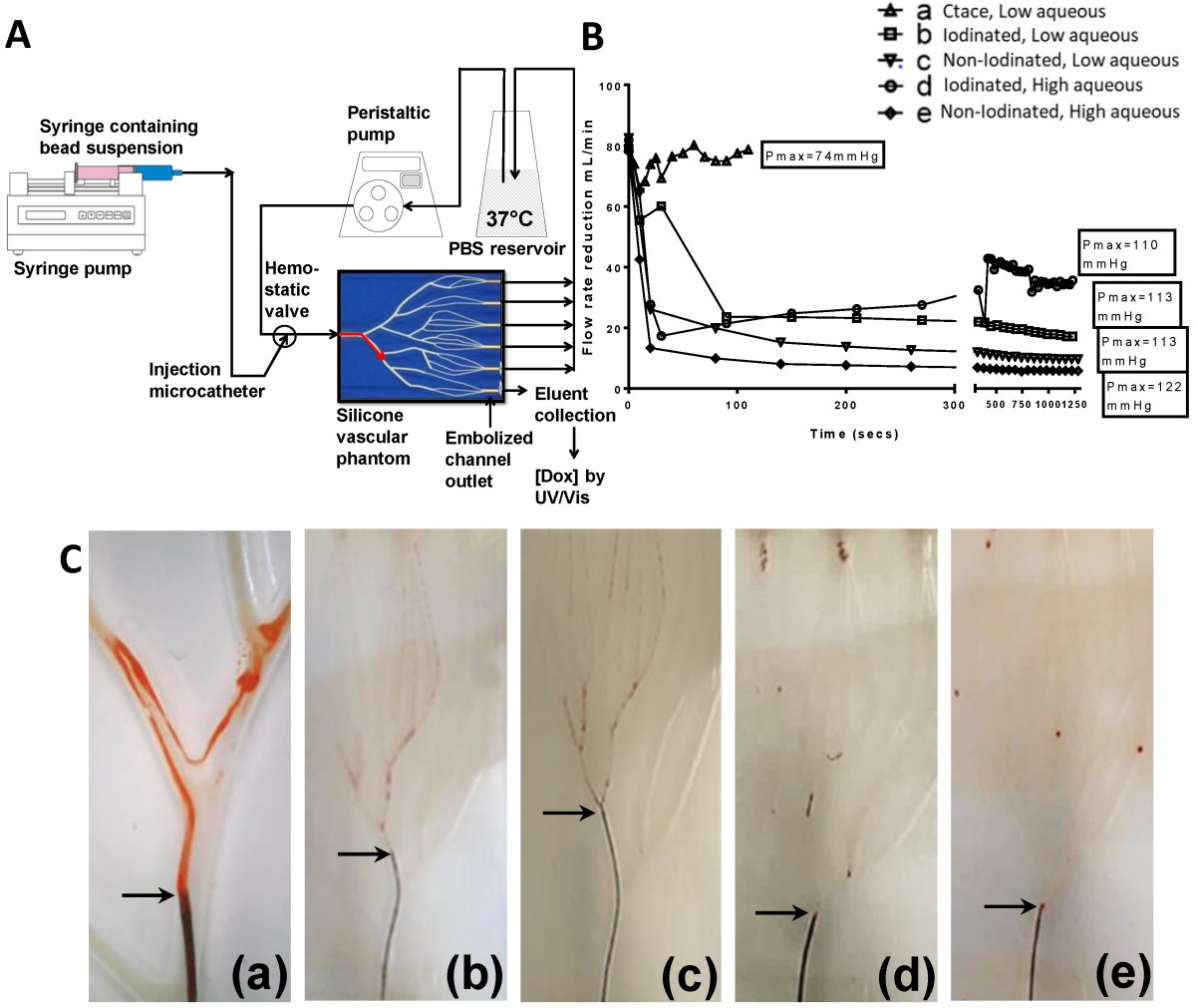
Combined *in vitro* test setup (A) *in vitro* emulsion flow rate reduction and hydrostatic pressure maximum (B), *in vitro* dynamic flow profiles (C), defined as; cTACE (a), Non-Iodinated low aqueous (b), Non-iodinated high aqueous (c), Iodinated high aquous (d) and Iodinated low aqueous (e). Still images extracted from full videos available in S1.

The emulsion drop-test analysis reinforced the occurrence of phase partitioning for non-iodinated DEBs observed in the optical microscopy, again showing the beads separate from the poor stability emulsions upon dilution in an aqueous continuous phase (Figure 5A). Iodinated DEBs remained in the oil phase, stabilizing the oil as descrete, intact droplets as they entered the aqueous continuous phase (Figure 5B); this is more reminiscent of a cTACE emulsion behavior. The dynamic stability of emulsions was assessed in terms of droplet formation under flow following release from the catheter tip under various injection rates using a Vascular Flow Model (VFM, Figure 5C 20). Droplet size is influenced by flow and shear rates in the delivery channel upon administration into the continuous aqueous phase 23. Through this method it was possible to identify development of droplets and their integrity as they flowed through a series of bifurcations. This was used to identify formulations that would be robust and stable for use in pre-clinical testing. VFM droplet stability was consistently higher in iodinated bead containing emulsions compared against non-iodinated bead (Figure 5 D&E). Selection of “best case” conditions was based on a scored 1-5 index for each of the test parameters relating to their influence on clinical performance (Table 2). Iodinated DEBs showed the widest stable formulation range, with the lowest recorded flow stability for iodinated DEBs being observed with those formulations containing a higher aqueous content (Figure 4). The DEB emulsions demonstrate a clear ability to progressively occlude the flow in the VFM channels (with increasing hydrostatic pressure) due to the embolic effect of the particle, compared to the cTACE emulsion where Lipiodol™ passed freely through the 25 µm VFM filter ports without any occlusive effect (Figure 6B). This is aligned with both the established literature 8, 24 and the proposed mechanistic action of the novel emulsions 25. In clinical practice cTACE would be followed by some form of embolic particle, however it is worth noting that prior to embolisation the systemic drug release profile shown *in vitro* would be experienced *in vivo.*

### Drug elution *in vitro*

**Figure 7:**
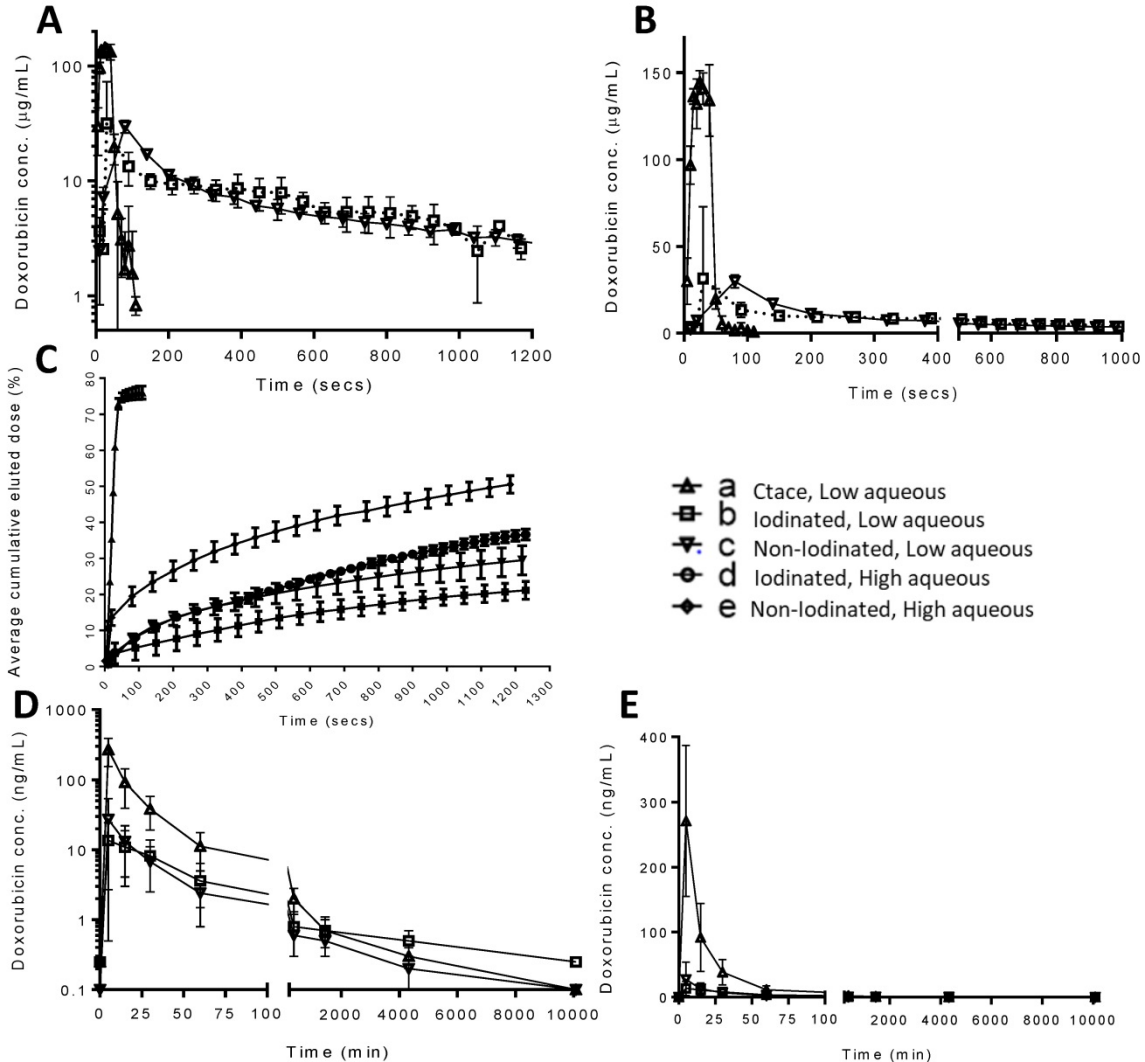
*In vitro* drug elution profiles for iodinated and non-iodinated low aqueous and cTACE low aqueous in log scale (A), linear scale (B) and percentage drug relase as a function of the total loaded drug amount including high aqueous emulsions (C). Corresponding *in vivo* PK release in VX2 rabbit model, for iodinated and non-iodinated low aqueous and cTACE low aqueous presented in log scale (D) and linear scale (E). Error bars represent standard deviation (n=3).

A modified VFM set-up was employed to profile the stability of drug-containing emulsions in terms of the drug elution properties of a selection of the previously characterised formulations (Figure 6A). The DEB emulsion formulations were based upon those tested in the stability assessment and were those highlighted in Figure 5, consisting of a low and high aqueous formulation of each bead type to evaluate the two extremes of the working stability range. The cTACE emulsion consisted of a 10mL Lipiodol™: 2mL aqueous Dox (25 mg/mL) mixture. The drug burst effect is ultimately associated with systemic free-drug and it is critical to understand the relationship between the emulsion stability and the *in vitro* pseudopharmacokinetic profile generated using the VFM. The low aqueous DEB emulsions, with increased level of droplet stability under flow conditions, showed slower Dox elution compared to their high aqueous DEB emulsion counterparts. All DEB emulsions show significantly slower release of drug compared to the cTACE (non-embolic) formulation (Figure 7).

Close examination of the emulsion flow through the VFM channels shows that the more stable low aqueous DEB emulsions remain as intact droplets throughout their journey from catheter tip to distal end-channel, where the bead-oil droplet accumulate without separation at the filter but still as discrete droplets that allow some residual flow (Figure 5C(d & e) and Supplementary videos S1). The relatively less stable high aqueous DEB emulsions showed a tendency to separate, particularly at bifurcations where higher shear forces are experienced, with the beads alone accumulating at the distal exit, whilst most the Lipiodol™ passed through the filter (Figure 6C(d & e)). Interestingly these emulsions reduce the residual flow more effectively and result in a slightly higher hydrostatic pressure (Figure 6B).

### Emulsion drug release, flow properties and imaging *in vivo*

#### Rabbit VX2 model for PK and radiopacity distribution

A rabbit VX2 tumour model was used to carry out a pharmacokinetic (PK) study of Dox release from the same formulations tested in the VFM. Figure 7A-C shows the *in vitro* pseudopharmacokinetic drug release profiles on a log scale for the emulsions tested in the VFM. These profiles clearly show the high quantity of Dox that is released as a burst in the first 100 seconds during administration of a cTACE emulsion (~150 µg/mL). This is in contrast to the very much reduced peak Dox concentrations (~25 µg/mL) that are observed with the DEB emulsions. These results compare very favourably with the findings of the PK study in the rabbit VX2 tumour embolization (Figure 7D(log) & E(linear)), where in this case the peak concentrations (C_max_) of Dox are observed at the first measured time point around 5 mins and are much greater for the cTACE treated animals (~275 ng/mL) compared to the much reduced C_max_ seen for the DEB Emulsions (15-30 ng/mL). In this study there was very little difference between the data measured for the iodinated *versus* the non-iodinated low aqueous DEB emulsions, both behaving similarly in terms of stability and handling during administration.

**Figure 8:**
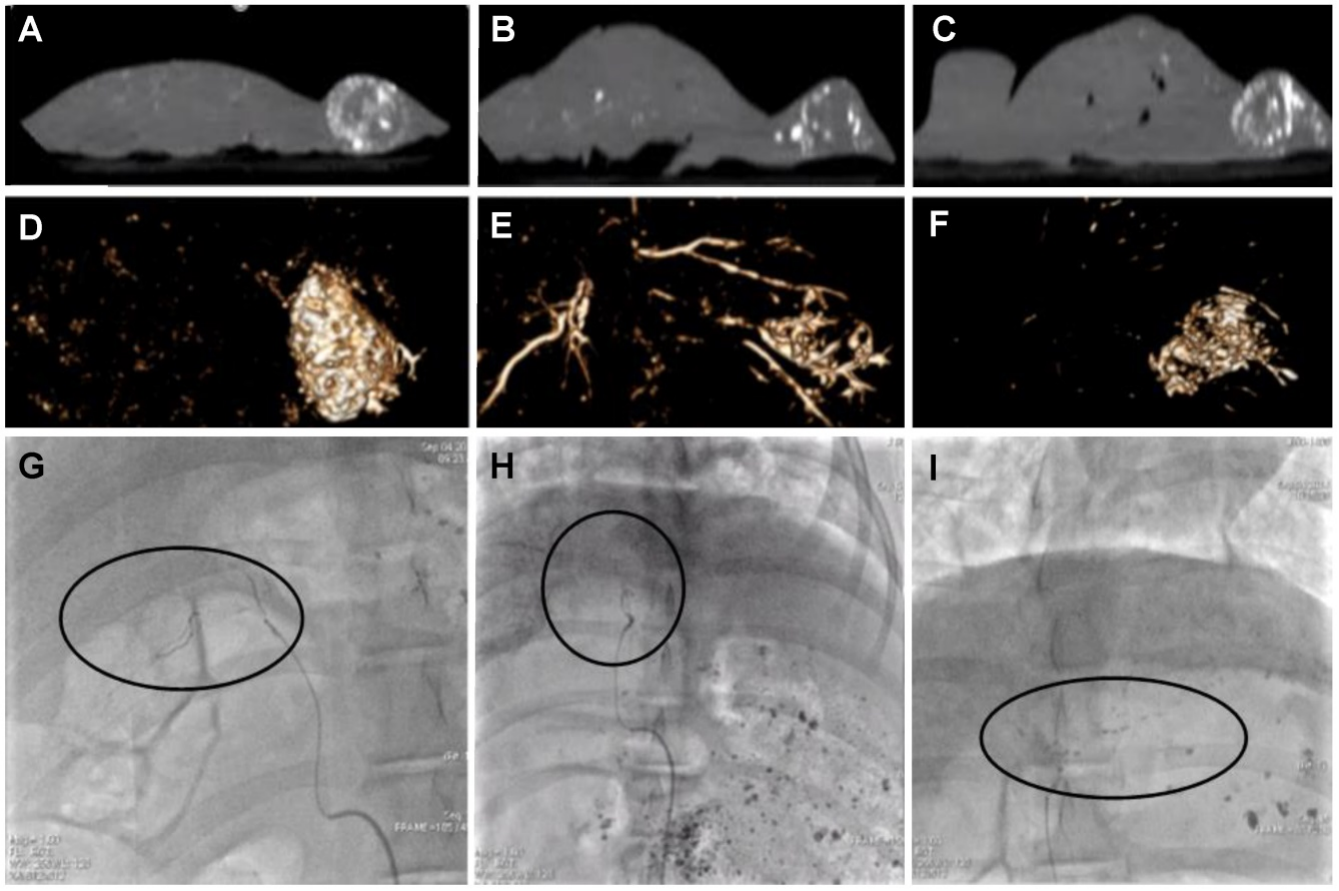
Single plane CT (Discovery IGS 730, GE Healthcare) slices showing emulsion residence after 7 days in VX2; cTACE (A), Non-iodinated (B) and Iodinated (C), with 2D volume reconstruction (D-F). Single representative angiographic images of flow characteristics for cTACE (H), Non-iodinated (G) and Iodinated (I). Black circles highlight flowing emulsions with clear droplet/ “packet-like” emulsions in cTACE (G) and Iodinated (I) but with loose more disperse flow in Non-iodinated (H) echoing flow characteristics observed *in vitro.* Still images extracted from full videos available in S1.

Animals treated with all formulations were survived for 7 days after which spatial distribution of the radiopaque components of the remnant emulsions in the liver was assessed under cone beam computed tomography (CBCT). Figure 8A-C show single slice images from the CBCT in the sagittal plane thresholded to remove the surrounding tissues that show the outline of the liver and the nodular mass on the right hand side of the organ that is the tumour. The white areas in this image are the radiopaque components that remain post-treatment with the various emulsions. Figure 8D-F shows a volume rendered image to portray the three-dimensional distribution of radiopaque matter. There was highly concentrated tumour filling of radiopaque matter in both cTACE and iodinated DEB emulsions (Figure 8A & C, D & F) indicating good tumour vessel filling. Non-iodinated DEB emulsions presented a more diffuse filling pattern, with some of the more proximal feeding vessels containing significant amounts of radiopaque material (Figure 8B & F). These emulsions also presented more difficulties in delivery *via* microcatheter with increased resistance and intermittent catheter clogging indicative of the stability issues encountered during *in vitro* testing. Consequently, only an average of 0.63 ± 0.15 mg of the 1mg planned dose could be delivered with the non-iodinated DEB emulsions compared to 0.95 ± 0.19 mg for the iodinated DEB emulsion. As shown in Figure 7D & E, the average plasma dose of Dox is higher for the non-iodinated emulsions (27.2 ± 26.7 ng/mL when compared to the iodinated DEB emulsion (13.6 ± 10.9 ng/mL) at the 5 minute time point, further illustrating the disparity in stability between the two emulsion composed of different DEB chemistries.

#### Swine handling and flow properties

A series of formulations were reconstituted with both iodinated and non-iodinated DEBs as described in Table 4. These included some emulsions formulated with smaller DEB size (40-90 µm) in order to assess their handling as these were used in the VX2 study due to the anatomic restrictions in vessel size. In general, non-iodinated DEB emulsions were ranked less stable in the syringe and handled more poorly during delivery compared to cTACE or the formulations using iodinated DEBs (Table 4). The *in vivo* stability however, appeared good under fluoroscopic imaging and the formulation tended to form droplets in the vessels more rapidly than the Lipiodol™-Dox cTACE mixture. The embolic effect was similar to cTACE but the occlusion was more proximal with the DEB-emulsion formulation compared to cTACE. The aspect of the product under fluoroscopy was also different: the formulation with the non-iodinated DEBs showed a more scattered deposition in the arteries compared to cTACE.

The stability in the syringe for all formulations reconstituted with iodinated DEBs was good. There was a strong effect of dilution on all the parameters assessed: increasing the dilution with contrast agent decreased the *in vivo* stability and the embolic effect of the formulation and increased the distality of occlusion. The performance of formulations with iodinated DEBs (containing between 4 mL and 6 mL of contrast agent) seemed to be close to the performances of cTACE. For the formulation with the lower dilution, the embolic effect seemed to be higher than for cTACE or formulations using non-iodinated DEBs. However after waiting a few minutes, flow was regained, more product could be injected and the total volume of formulation injected was in the same range as for cTACE and formulations with non-iodinated DEBs. The flow and visual appearance of the introduced emulsions echoed that of the VFM pre-selection testing and it was possible to identify droplets and string formations under fluoroscopy as shown in Figure 8G-I (Supplementary video S1).

## Discussion

All of the currently available commercial DEBs are composed of hydrogel materials that are substantially swollen in aqueous media and highly hydrophilic in nature. Mixing these directly with Lipiodol™ creates a incompatible concoction unsuitable for TACE consisting of an unstable multiphase system in which the DEBs reside in the aqueous phase. The rationale for the preparation of the novel DEBs used in this study was to modify the surface properties to make them more hydrophobic in an effort to encourage their accumulation at the oil:water interface and to help in the steric stabilization of the resulting emulsions. The non-radiopaque DEB based on DC Bead™ was lyophilized to create a porous structure that when initially mixed with Lipiodol™, would uptake the oil into part of the bead matrix. This has been used to impart radiopacity to the bead but we also surmised that the surface properties would also be rendered more hydrophobic in nature and hence more compatible in an subsequent oil:water emulsion. Note that the lyophilization process was shown not to alter the drug release kinetics of the beads (data not shown). The second approach was to employ the more hydrophobic polymer used to make DC Bead LUMI™ in which the DEBs are modified with hydrophobic triiodobenzyl groups in order to make them permanently radiopaque. Our testing of the lyophilized DEBs using AFM force-distance-amplitude measurements demonstrated that there was a greater pull-off force for the non-iodinated bead compared to the two iodinated beads tested. Since the AFM probe tip is hydrophilic and has a greater attractive force for more hydrophilic surfaces, the non-iodinated bead was determined to have a comparatively less hydrophobic surface than the iodinated beads; and similarly the 33 mg iodine/mL iodinated bead, had a greater pull of force compared to the 155 mg iodine/mL iodinated bead. Hence, the greater the iodine content, the greater the hydrophobicity of the surface and the lower the pull-off force measured.

An increased or comparable rate of drug loading was observed in DEB emulsions compared to DEBs loaded by the standard pharmacy methods. For the non-iodinated DEB emulsions there was a marked incease in loading rate with >98% Dox loaded within 2 mins compared to 30 mins for DC Bead™. This effect is due to a higher surface area to volume ratio of DEBs to Dox maintained in the water phase in which they both reside once the emulsion is formed. The addition of iodine to the structure of the iodinated DEBs increases surface hydrophobicity, causing DEBs to partition into the oil phase, thus slowing the loading rate by restricting access to the drug that is partitioned in the aqueous compartment of the emulsion. Loading rates for Dox in this instance are comparable between the iodinated DEB emulsion and standard methods used for drug loading DC Bead LUMI™. It is interesting that in clinical practice at high volume centres or where there is a need to rapidly prepare another drug loaded vial of DEB for immediate administration, the non-iodinated DEB emulsion formulation offers an almost instantaneous process of loading the DEBs with high levels of Dox without the need to wait.

Microscopic analysis of the various DEB emulsions revealed that it was possible to formulate the two DEB types in order to enhance preference for DEBs to reside at the oil:water interface and stabilise the droplets. This is achieved however, in a quite different manner for the two DEB types as the non-iodinated DEB has a preference for the aqueous phase, where as the iodinated DEB has a preference for the Lipiodol™ oil phase. This is despite the fact that the non-iodinated DEB is mixed with Lipiodol™ in the first instance in order to absorb it into its porous structure, prior to mixing with the aqueous component. This means the surface of the non-iodinated DEB remains more hydrophilic in character. 26. Particles can be used to stabilize emulsions (Pickering emulsions) but these are typically formed using small particulates in the range 200 nm- 1µm, as small particle sizes give high packing efficiency at the oil:water interface 27. Here DEBs in the size range 40-150 μm have been used in the creation of various emulsion formulations, that whilst not adding to the stability of emulsions relative to the non-DEB containing emulsion, are sufficiently resistant to separation to allow their practical use in a TACE procedure. The more hydrophobic radiopaque DEBs appear to provide a far superior stability compared to the non-radiopaque DEBs, suggesting their presence within the oil phase and at the oil:water interface, is more efficient in helping to retard the separation of the phases.

The stability of an emulsion directly relates to its handling and administration through a microcatheter and hence suitability for use in TACE procedures. Unstable emulsions form a clear separation of phases within a short timeframe (<5mins) at which point delivery becomes difficult to achieve. The visual appearance of the emulsions can also indicate its type, (w/o, o/w) under dye assisted light microscopy and whether there has been an inversion of the phases, which is important from a drug delivery perspective if the aqueous drug is no longer confined within the continuous oil phase. In a study by de Baere et al. 28, an *in vitro* comparison using a variety of characterisation methods was conducted under shear and pressure rates representative of those experienced in large human arteries 28. As part of this investigation de Baere et al. utilised an *in vitro* “hepatic arterial tree” composed of an 8 mm bifurcating silicon tube with a continuous phase of glycerol at 45%, for characterisation of droplet mixing and emulsion type 21. An evolution of this platform termed the Vascular Flow Model (VFM) was used in this study for the characterisation of DEB emulsions under dynamic flow conditions. DEB emulsion stability under flow was consistently higher in iodinated DEB emulsions compared to the non-iodinated DEB versions, although stability decreased markedly for both formulations as the content of the aqueous component was increased. This is believed to be due to the tendency for the o/w emulsion to invert to a w/o state, increasing the surface area of the aqueous compartment and diluting the DEB stabilizing effect, which increases dispersion and breakdown of the emulsion within the flowing aqueous continuous phase. The DEB emulsions were obviously capable of being trapped at the channel outlet by the filter and accumulated at the distal end causing a drop in flow rate in the channel and a subsequent increase in the hydrostatic pressure in the system due to this occlusion. The non-radiopaque DEB emulsions were more effective in blocking the flow as these beads are somewhat softer than the iodinated counterparts as we have previously shown 29 and pack together at the distal end more effectively by reducing the space between the particulates, whereas the iodinated DEBs being very stiff, accumulate but remain as spherical spheres with significant inter-sphere spaces present to allow some residual flow and relief of the pressure (Figure 6B).

As the Dox was shown to prefentially load from the aqueous componment of the mixture into the DEBs in the formulation, the DEB emulsions clearly demonstrated a similar controlled Dox release and low “systemic” burst of drug that is synonymous with DEB-TACE compared to cTACE 8, 30. The more stable iodined DEB low aqueous formulation produced the slowest release profile, which is expected if the drug has to diffuse out of the DEB and cross the oil boundary before being released into the flowing continuous phase and *vice versa* for the less stable emulsions with higher aqueous content. The pseudopharmacokinetic profile generated using the VFM was a reasonable prediction approximation to the actual PK profile measured in the rabbits in terms of general overall shape, peak drug times both occurring early on in the first few minutes, and both reporting similar trends in drug release from the three emulsions investigated. A major limitation of the VX2 model is the reduced vasculature dimensions when compared to that of human hepatic vasculature 31. This means that administered doses are scaled according to body weight and vascular access, resulting in scaled representations of the dosimetry experienced in a procedure 31. This was observed to be particularly impactful in the delivery of emulsions, where difficulties administering the full emulsion dose calculated as part of the agreed protocol were experienced. In these cases it was recorded and included as feedback for emulsion evaluation in terms of handling and overall grade. This further highlights the importance of channel geometry and size for *in vitro* profiling experiments. This should ideally always be performed using flow conditions that closely replicate those present within the intended treatment indication.

The results generated here demonstrate the benefit of *in vitro* devices in the evaluation of novel emulsions. Whilst the VFM does not emulate the same level of flow dynamic complexity as the *in vivo* situation, it does provide a profiling step that enabled selection of favourable iodinated and non-iodinated DEB emulsion formulations suitable to carry forward for *in vivo* testing. Visualization of flow of all of the emulsions tested using fluoroscopy was clear, as all emulsions either contained Lipiodol™, contrast agent or radiopaque DEBs, all of which are radiodense and will be visible under fluoroscopic X-ray. Radiopaque DEB emulsions could be delivered as descrete imageable droplets, whereas the non-radipaque DEB formulations seemed to occlude the arteries more proximally which could be related to poor emulsion instability as noted *in vitro*. Lilienberg et al. reported similar conclusions in their swine study, where they observed that the physical embolic effect of emulsions were directly related to frequency of mixing and the stability under flow 24. After the treatment of the rabbit VX2 tumours, the animals were survived for seven days before imaging. This allows for any trapped contrast agent to be washed out of the vessels 32 and for Lipiodol™ in healthy parts of the liver to be cleared by phagocytosis in the Kupffer cells 33. CBCT showed more compact densification of the tumour in the cTACE and iodinated DEB emulsion cases indicative of improved penetration into the tumour vessels. The radiopacity in the DEB emulsion case could be due to the presence of both radiopaque beads and Lipiodol™ in the tumour but it is not possible to distinguish between the two in this instance as CBCT is not sensitive enough to detect at single bead resolution. The non-iodinated DEB emulsion was less well-filled and several more proximal feeding vessels obviously contained what is likely to be beads, indicating potential separation of the formulation during delivery.

To our knowledge, this is the first time that novel DEBs have been prepared that are capable of being formulated into stable DEB emulsions with Lipiodol™ and Dox that are suitable for application in TACE procedures. These emulsions therefore potentially combine the advantages of portal vein filling observed with Lipiodol™-based TACE with the beneficial safety profile of the controlled and sustained drug delivery obtained using DEB-TACE. *In vitro* profiling supports adequate stability for handling and administration and *in vivo* evaluation confirms low peak drug plasma levels and prolonged radiopacity of the treated area showing the bead+Lipiodol™ location. Further refinement and in vitro evaluation combined with efficicacy testing *in vivo* of this promising combined technology platform is warranted.

## Supplementary Material

Supplementary figures and tables.

## Figures and Tables

**Table 1:** Showing AFM force adhesion results for each bead chemistry.

Sample	Average pull-off force (mV)
Non-iodinated Bead (0mg/mL)	0.8 ± 0.05
Iodinated Radiopaque Bead (33mg/mL )	0.2 ± 0.1
Iodinated Radiopaque Bead (155mg/mL )	0.1 ± 0.05

**Table 2:** Showing quality attributes for each rank of emulsion.

Rating	Drop Test	Dynamic Flow Test	Separation Time (Mins)
*	Loose DEBs with stream of oil.Few DEBs associated with the oil.	Stream of loose DEBs. Oil and DEBs completely separate. Immediate separation of DEBs from oil.	<1
**	Stream oil with some DEBs associated with the oil but mostly loose DEBs.	Some stream of oil and DEBs together. Few DEBs at the oil/aqueous interface. DEBs separate quickly.	2-3
***	Stream of oil with DEBs mainly associated with the oil. Droplet formation with DEBs within the droplet and migration to the aqueous interface or significantly on the outside. Oil droplets disintegrate rapidly.	Stream of oil and DEBs together.Formed droplets or stream of oil containing DEBs. DEBs significantly at the oil/aqueous interface. Separation of DEBs at bifurcation and during passage.	3-5
****	Mainly oil droplets formed with streams of oil with DEBs. Droplet formation with DEBs mainly within the droplet. DEBs within stream are visually bound within the oil steam. Oil droplets sometimes hold together.	Mainly formed droplets with DEBs that may be at oil/aq. interface. Good retention of DEBs with limited separation of DEBs at bifurcation or during passage.	5-10
*****	Mainly oil droplets formed with streams of oil with DEBs. DEBs within stream are visually bound within the oil steam. Oil droplets hold together.	Formed discreet droplets. DEBs may be at oil/aq. interface but maintained in oil droplets. Good retention of DEBs with minimal separation at bifurcation or during flow.	>10

**Table 3:** Showing allocated rank for each test emulsion in terms of oil: aqueous composition and bead chemistry. This is the sum of the performance of droplet test, flowing emulsions and general syringe stability.

Composition (% v/v)					Emulsion Rating	
Formulation	Total Oil	Total Aqueous	Aqueous Mix		Non-Iodinated	Iodinated
			Dox Solution	Contrast Agent		
1	52.6	47.4	31.6	15.8	+	+ + +
2	55.6	44.6	11.1	33.3	+	+ + +
3	55.6	44.4	22.2	22.2	+	+ + +
4	58.8	41.2	23.5	17.7	+	+ + + +
5	58.8	41.2	17.7	23.5	+	+ + + +
6	58.8	41.2	2.9	38.3	+	+ + + + +
7	62.5	37.5	25.0	12.5	+ +	+ + + + +
8	62.5	37.5	12.5	25.0	+ +	+ + + + +
9	66.7	33.3	13.3	20.0	+ + +	+ + + + +
10	69.0	31.0	7.7	23.3	+ + +	+ + + + +
11	69.0	31.0	18.1	12.9	+ + +	+ + + + +
12	71.4	28.6	14.3	14.3	+ + +	+ + + + +
13	71.4	28.6	7.2	21.4	+ + +	+ + + + +
14	76.9	23.1	15.4	7.7	+ + +	+ + + + +
15	83.3	16.7	4.2	12.5	+ +	+ + + + +

**Table 4:** Showing the range of test emulsions incorporated into the swine flow and handling study to cover the working range identified *in vitro* with allocated rankings and bead chemistry.

Formulation	Bead		Emulsion Mix			Rank
	Type	Volume/Bead Size	Lipiodol	Dox	Contrast	
A	Non-iodinated	2mL / 70-150µm	10mL (74%)	2mL (15%)	1.5mL (11%)	**
B	Iodinated	1.5mL / 70-150µm	10mL (63%)	2mL (13%)	4mL (26%)	*****
C	Iodinated	1.5mL / 70-150µm	10mL (50%)	2mL (10%)	8mL (40%)	****
D	Non-iodinated	2mL / 40-90µm	10mL (74%)	2mL (15%)	1.5mL (11%)	**
E	Iodinated	1.5mL / 40-90µm	10mL (63%)	2mL (13%)	4mL (26%)	*****
F	Iodinated	1.5mL / 70-150µm	10mL (55%)	2mL (11%)	6mL (33%)	****
cTACE	-	-	5mL (67%)	2.5mL (33%)	-	*****
